# Risk factors and outcomes of infants with necrotizing enterocolitis: a case-control study

**DOI:** 10.3389/fped.2025.1611111

**Published:** 2025-09-10

**Authors:** Noela H. B. Kamanga, Reenu Thomas, Kebashni Thandrayen, Sithembiso C. Velaphi

**Affiliations:** Department of Paediatrics and Child Health, Chris Hani Baragwanath Academic Hospital, Faculty of Health Sciences, School of Clinical Medicine, University of the Witwatersrand, Johannesburg, South Africa

**Keywords:** neonate, necrotizing enterocolitis, sepsis, risk factors, outcomes

## Abstract

**Introduction:**

Necrotizing enterocolitis (NEC) is a condition associated with high mortality and morbidity. Its pathogenesis is linked to intestinal immaturity, inflammation, and enteral feeding. Identifying risk factors for the development of NEC and its mortality can inform targeted preventative strategies.

**Aim:**

The aim of the study was to assess the incidence, characteristics, risk factors, and outcomes of infants diagnosed with NEC in a large tertiary neonatal unit in South Africa.

**Methods:**

A prospective case-control study was conducted from May 2022 to December 2024 at Chris Hani Baragwanath Academic Hospital. Infants diagnosed with definite NEC (modified Bell's stage 2 or 3) were included as cases. Each case was matched with 1–2 controls by weight and postnatal age. The demographic characteristics, laboratory findings, management, and outcomes of cases and controls were reviewed. Comparisons were performed between cases and controls, and between survivors and non-survivors amongst the cases using univariate and multivariate logistic regression analyses.

**Results:**

There were 167 cases of NEC enrolled. The incidence rate of definite NEC was 3.4/1,000 live births, comprising 1.4% and 4.0% of all neonatal admissions and very low birth weight infants, respectively. The median gestational age, birth weight, and postnatal age of cases were 31 weeks, 1,455 g, and 8.5 days, respectively. Cases were more likely to have been formula fed (OR: 2.00; 95% CI 1.20–3.33), have been previously exposed to a longer duration of antibiotics (OR: 1.26; 95% CI 1.14–1.40), and to have received a blood transfusion (OR: 27.4; 95% CI 2.09–359), and less likely to have reached full feeds in a shorter time (OR: 0.88; 95% CI 0.80–0.95). In total, 91 cases (54.5%) had culture-confirmed sepsis. The mortality rate was 49.7%, with ventilation and hypotension predictors of mortality.

**Conclusion:**

There was a high incidence of definite NEC, with associated high mortality, mainly in infants who were ventilated and hypotensive. Factors associated with NEC were formula feeding, longer duration of antibiotics, and prior blood transfusion.

## Introduction

Necrotizing enterocolitis (NEC) is a devastating disorder of the gastrointestinal tract in neonates, characterized by inflammation, ischemia, and infection (1). There is resultant necrosis of the bowel and, in severe cases, perforation (2). This condition results in significant morbidity and mortality and is the most common surgical emergency affecting neonates (3). The incidence rate has been reported as 1.26 per 1,000 live births in South African neonatal units, comparable to that of 1–3 per 1,000 live births globally (4, 5). It has been reported to occur in 2%–9% of very low birth weight infants and is associated with a high mortality rate, ranging from 20% to 50% (1, 4, 6). The diagnosis is made based on clinical features, and confirmed on plain abdominal radiography and, increasingly, abdominal ultrasound scan. Grading is conducted according to Bell's modified criteria, with stage 1 indicating suspected disease, stage 2 definite disease, and stage 3 advanced disease (7, 8). Treatment includes bowel rest, parenteral nutrition, and antibiotics, and severe cases may require surgical intervention, respiratory support, and inotropes (1, 9).

Prematurity is a major risk factor for this condition, with approximately 85% of cases occurring in preterm infants (1). NEC is most likely due to poor motility, an immature barrier, and the immune functions of the preterm intestinal tract (3). Most preterm infants have prolonged hospitalization and are often on antibiotics, resulting in dysbiosis (an imbalance between the intestinal flora and pathogenic bacteria) (9, 10). Exclusive human milk feeding significantly decreases the risk of developing NEC when compared to formula feeding (11). Human milk contains Bifidobacteria, immunoglobulins, lactoferrin, oligosaccharides, and other bioactive components that are protective (3, 12). Although the above risk factors have been described, the pathophysiology is still incompletely understood. Other risk factors that have been implicated are infection, blood transfusion, patent ductus arteriosus, or any event that exposes the neonate to hypoxia or ischemia. Factors associated with NEC vary in different reports, and most of these reports are retrospective studies, which have a high risk of bias (13–16).

The main objectives of this study were to determine the incidence, risk factors, and associated mortality in infants diagnosed with definite NEC who were hospitalized at the Chris Hani Baragwanath Academic Hospital (CHBAH) neonatal unit, a large public tertiary academic hospital in Johannesburg, South Africa, by conducting a prospective case-control study.

## Materials and methods

This was a prospective case-control study that included infants diagnosed with definite NEC who were admitted to the neonatal unit and matched controls. All neonates diagnosed as having definite NEC who were hospitalized and managed between 1 May 2022 and 20 December 2024 were eligible as cases.

### Study setting

Cases included any infant diagnosed as stage 2 or 3 NEC according to Bell's modified criteria (7). NEC cases diagnosed at CHBAH and NEC referrals from other hospitals were included. For the cases diagnosed at CHBAH, two controls were matched according to birth weight (±100 g) and postnatal age (±7 days). The NEC cases referred from other hospitals were not matched with controls. Infants with congenital intestinal abnormalities or severe congenital abnormalities were excluded from both the case and control groups.

CHBAH and local clinics conduct approximately 30,000 deliveries with an average of 4,000 admissions to the CHBAH neonatal unit per year. The neonatal unit has 185 beds, of which 18 are neonatal intensive care beds. Preterm deliveries account for 20% of all deliveries (17). The hospital is a major surgical referral center for the surrounding district and regional hospitals as it is one of the few hospitals offering pediatric surgical services in the Gauteng and North West provinces.

### Data collection

The research team was informed by the clinical team at CHBAH of any newly diagnosed NEC cases. Diagnosis of definite NEC was based on a clinical diagnosis by the attending pediatrician, which is based on the presence of gastrointestinal tract symptoms, abdominal distension, and radiologic findings of pneumatosis intestinalis with or without signs of intestinal perforation. The parents of both cases and controls were approached for written informed consent for inclusion in the study. Each participant was assigned a case or control number, and their data were entered into an anonymized data sheet and then into the Research Electronic Data Capture (REDCap) database.

Baseline maternal data were collected, including demographic characteristics, human immunodeficiency virus (HIV) status, presence of medical conditions, and maternal medication, including magnesium sulfate (MgSO_4_), antenatal steroids (ANS), and antibiotics. The collected infant data included demographic characteristics, delivery mode, Apgar score, and need for resuscitation at birth. Data regarding the feeding of the infants were collected and included time to first feed; type of feeding; whether fed human milk (mother's own milk or donor breast milk), formula, or both; administration of FM85 milk fortification; and time to full enteral feeds. Other information collected included the medical management of controls and cases before NEC diagnosis, which included the use of antibiotics, blood transfusion, inotropes, mechanical ventilation, and other diagnoses, namely patent ductus arteriosus (PDA). Detailed information regarding all NEC cases was collected, including age at diagnosis, stage of disease, laboratory results, presence of infectious organisms, type of surgical management, and outcomes. Management, such as ventilation, the use of inotropes, and the type of antibiotics, was documented.

### Statistical analysis

The statistical analysis was performed using TIBCO Statistica® 14.0.0.15. For the identification of risk factors for NEC, only the cases diagnosed at CHBAH and their controls were analyzed. For the descriptive analysis, all cases were analyzed, including NEC cases referred from other hospitals. Continuous data were presented as means and standard deviations (SD) if normally distributed and medians with interquartile ranges (IQR) if not normally distributed. Categorical variables were presented as frequencies and percentages. When comparing the cases and controls or survivors and non-survivors, a chi-square test or Fisher's exact test was performed for categorical variables; and Student’s *t*-test or the Mann–Whitney *U*-test for continuous variables, based on whether they were normally distributed or not. Univariate and multivariate logistic regression analyses were performed to determine the risk factors for NEC, and these were reported as crude or adjusted odds ratios (ORs) with 95% confidence intervals (CIs). The variables included in the multivariate analysis were those with *p*-values < 0.10; thereafter, *p*-values < 0.05 were considered to be statistically significant. All the statistical tests were two-sided. Where data were missing for a case or matching controls, that case or control was removed from the analysis for that variable.

### Ethics

Permission to conduct this study was obtained from the hospital protocol review committee and ethics approval was provided by the University of the Witwatersrand Human Research Ethics Committee (Protocol number M220215).

## Results

### Incidence

**Figure 1:**
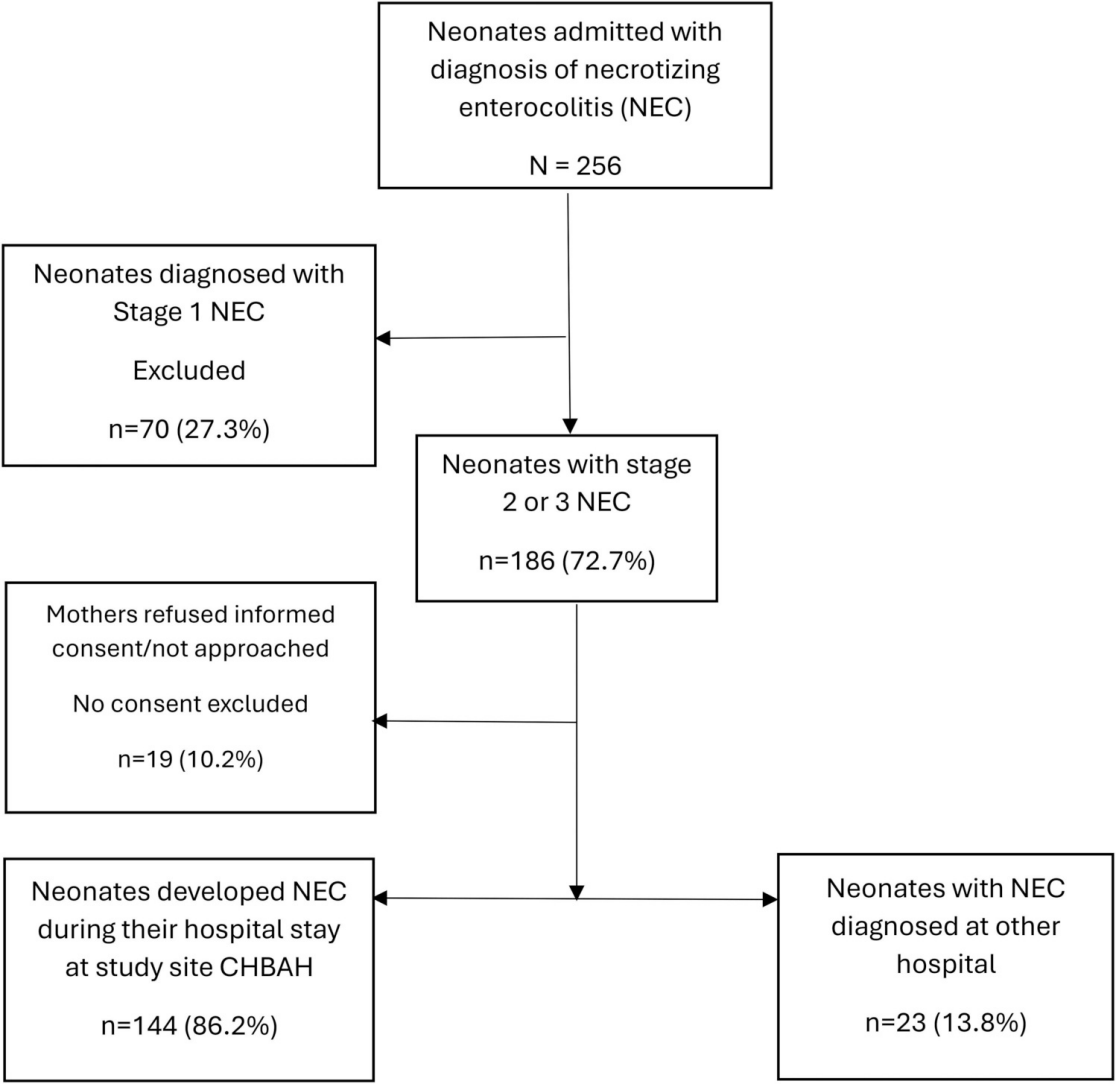
Flow chart of the number of patients diagnosed with necrotizing enterocolitis enrolled in the study.

There were 256 neonates admitted and diagnosed with NEC between 1 May 2022 and 31 December 2024. Stage 1 was diagnosed in 70 infants and these were therefore excluded, leaving 186 with definite NEC. Of the initial 186 definite cases, 160 were diagnosed at CHBAH, and 26 were diagnosed at other facilities (referrals). In total, the parents of 19 (10.2%) cases refused consent; thus, 167 cases were included in the study (Figure 1). Among the enrolled cases, a total of 144 (86.2%) cases were diagnosed at CHBAH and 23 (13.8%) were diagnosed at another facility and referred to CHBAH for surgical services. During the study period, there were 42,413 live births and 11,108 neonatal admissions, of which 1,886 were very low birth weight (VLBW). The incidence rate of NEC at CHBAH was 3.8/1,000 live births, comprising 1.4% of all neonatal admissions and 4.5% in VLBW infants (Table 1).

**Table 1:** Incidence of NEC according to live births, admissions, and very low birth weight infants.

Parameter	Number of live births	All admissions	Admissions of very low birth weight infants (birthweight <1,500 g)
Total number	42,413	11,108	1,886
NEC cases	160	160	85
Incidence	3.8/1,000 live births	1.4%	4.5%

### NEC patients (cases)

Of the 167 cases, 101 (60%) were assessed as stage 2 and 66 (40%) as stage 3 NEC (Table 2). The majority (62.3%) were managed medically, with the rest managed surgically. The median age at diagnosis was 8 days (IQR 5–16). The all-cause mortality rate was 47.9%, with the median age at death being 41 days (IQR 20.5–62). In total, 58 (35.4%) cases were hypotensive and required intravenous inotropes.

**Table 2:** Demographic characteristics, severity, management, and outcomes of neonates with NEC (*N* = 167).

Baseline characteristics	Number (%)
Birth weight (g)	1,310 (1,045–1,660)^a^
Gestational age (weeks)	31 (29–33)^a^
Male sex	95 (56.9)
Apgar scores	9 (8–10)^a^
Age at diagnosis (days)	8 (5–16)^a^
Modified Bell's staging	
Stage 2a	54 (32.3)
Stage 2b	47 (28.1)
Stage 3a	15 (9.0)
Stage 3b	51 (30.5)
Type of management	
Non-surgical management	104 (62.3)
Surgical management	63 (37.7)
Type of non-surgical management post-diagnosis	
Hypotension requiring inotropes	58 (34.7)
Mechanical ventilation	88 (52.7)
Blood transfusion	62 (37.1)
Type of surgical management	*N* = 63
Peritoneal drain inserted	20 (31.7)
Subsequent laparotomy	16/20 (80)
No subsequent laparotomy	4/20 (20)
Laparotomy	43/63 (68.3)
Outcome	
Number died	80 (47.9)
Median age at death (days)	41 (20–62)^a^

^a^ Median (interquartile range).

Among the 63 NEC cases that required surgical intervention, 20 (31.7%) were initially managed with peritoneal drain insertion, with 16 (80%) of these subsequently underwent a laparotomy. Among the cases that had an initial peritoneal drain and then underwent a laparotomy, 3 (18.8%) had primary anastomosis, 10 (62.3%) had an ileostomy/colostomy, and 3 (18.8%) had temporizing surgery (clip and drop) but died before definitive surgery could be performed. Among the 43 (68.3%) cases that directly underwent a laparotomy, 3 (7.0%) had primary anastomosis, 22 (34.9%) had an ileostomy/colostomy, and 14 (32.6%) had temporizing surgery (clip and drop). Among those who had the temporizing surgery, eight died before definitive surgery could be performed, and the remainder underwent definitive surgery. Among all those who underwent a laparotomy, two patients (2/59; 3.4%) were assessed to have NEC totalis.

The majority of cases (103; 64.8%) had normal white cell counts (WCCs), 50 (31.4%) had leucopenia and the remainder had leukocytosis (Table 3). Neutropenia was observed in 30 cases (20.5%) and 34 cases (21.4%) had thrombocytopenia. Culture-confirmed sepsis occurred in 54.5% of the cases, and the common organisms were *Klebsiella pneumoniae* (*n* = 41; 42.2%), *Acinetobacter baumannii* (*n* = 16; 16.5%), *Enterococcus* spp. (*n* = 14; 14.4%) and *Escherichia coli* (*n* = 6; 6.1%).

**Table 3:** Laboratory findings in infants with necrotizing enterocolitis.

Laboratory parameters	Number (%) or median (IQR)
Full blood count	*N* = 159
White cell count (WCC) (10^9^/L)	7.2 (3.9–11.8)^a^
Leucopenia (WCC < 5.0)	50 (31.4)
Normal (5–25)	103 (64.8)
Leukocytosis (>25)	6 (3.8)
Neutrophils (10^9^/L) (*N* = 146)^b^	3.56 (1.6–6.0)^a^
Neutropaenia (<1,500)	30 (20.5)
No neutropaenia) (>/ = 1,500)	116 (79.5)
Hemoglobin (g/dl)	13.0 (10.5–15.1)^a^
Platelets (10^9^/L)	225 (123–337)^a^
Thrombocytopenia (<100)	34 (21.4)
No thrombocytopenia (>/ = 100)	125 (78.6)
C-reactive protein^b^	*N* = 158
<10 mg/L	66 (41.8)
≥10 mg/L	92 (58.2)
Blood cultures	
Culture confirmed sepsis at diagnosis (*n*, %)	91 (54.5)
Organisms cultured (*n* = 97)	
*Klebsiella pneumoniae*	41 (42.2)
*Acinetobacter baumannii*	16 (16.5)
*Enterococcus* spp.	14 (14.4)
*Coagulase-negative staphylococcus*	8 (8.2)
*Escherichia coli*	6 (6.1)
*Candida auris*	4 (4.1)
*Enterobacter cloacae*	2 (2.0)

^a^ Median (interquartile range).

^b^ Neutrophil counts were missing for 13 infants and one C-reactive protein result was missing.

### Comparing cases and controls

There were 144 cases that developed NEC at CHBAH (born and admitted at CHBAH/local clinics). These were matched with 1–2 controls, with a total of 206 enrolled controls. There were no significant differences in the maternal characteristics between the cases and controls. Maternal age and frequency of comorbidities, such as hypertension and diabetes mellitus, were similar between the two groups (Table 4). Though the cases were matched with controls (± 7 days), the cases had a higher postnatal age than the controls [8.5 days (IQR 16–16.5) vs. 8 days (IQR 3–15); *p* = 0.011]. Moreover, 88% of the cases were inborn, compared to 91.7% of the controls, but this was not statistically significant (*p* = 0.413). There were no statistically significant differences in gestational age, birth weight, Apgar score, or delivery room temperatures between the two groups. The cases had lower weight gain after recruitment compared to the controls [7.5 g/kg/day (IQR 0–12) vs. 13 g/kg/day (IQR 10–18); *p* < 0.001].

**Table 4:** Comparing the baseline characteristics of the NEC cases and the controls.

Characteristics	Cases	Controls	*P*-value
	*N* = 144	*N* = 206	
Maternal characteristics			
Maternal age, years, median (IQR)	29 (23–34.5)	29.5 (23–35)	0.562
Maternal positive HIV status, *n* (%)	46/142 (32.3)	61/206 (29.6)	0.580
Maternal diabetes, *n* (%)	4/144 (2.8)	6/206 (2.9)	0.940
Maternal hypertensive disorders, *n* (%)	24/144 (16.6)	31/206 (15.0)	0.682
Antenatal antibiotics, *n* (%)	13/144 (9.0)	14/206 (6.8)	0.444
Antenatal steroids, *n* (%)	80/143 (55.9)	106/206 (51.4)	0.408
Antenatal MgSO_4_, *n* (%)	24/144 (16.7)	48/206 (23.3)	0.127
Delivery by cesarean section, *n* (%)	77/143 (53.8)	122/205 (59.5)	0.107
Multiple gestation, *n* (%)	28/144 (19.4)	46/206 (22.3)	0.513
Infant characteristics			
Birth weight, gr, median (IQR)	1,450 (1,165–1,692)	1,450 (1,195–1,665)	0.940
Gestational age, weeks, median (IQR)	31 (29–33)	31 (29–33)	0.930
Postnatal age at enrolment, days, median (IQR)	8.5 (6–16.5)	8 (3–15)	0.011
Male sex, *n* (%)	81 (56.2)	102 (49.5)	0.231
Apgar, median (IQR)	9 (8–10)	9 (8–10)	0.402
Delivery room temperature, ℃, median (IQR)	36.0 (35.0–36.0)	36.0 (35.0–36.0)	0.760
Need for resuscitation at birth	24/144 (16.6)	23/206 (11.1)	0.140
Weight gain before diagnosis (g/kg/day)	0 (−15–1.5)	−4 (−15–2)	0.494
Weight gain after diagnosis (g/kg/day)	7.5 (0–12)	13 (10–18)	<0.001

IQR, interquartile range; MgS0_4_, magnesium sulfate.

In the multivariate analysis, the neonates who had a longer time (days) to initiate feeding were less likely to develop NEC by 12% (OR-0.88; 95% CI 0.80–0.95; *p* = 0.003) and when comparing those who received any amount of breastmilk vs. those who were exclusively formula fed, there was a 50% reduction in developing NEC. The likelihood of having NEC was higher if the neonates were fed formula compared to breastmilk feeding (any volume) (OR 2.00; 95% CI 1.20–3.33; *p* = 0.008), when having received a longer duration of previous antibiotics (OR 1.26; 95% CI 1.14–1.40; *p* < 0.001), and when having received a blood transfusion (OR 27.4; 95% CI 2.09–359; *p* = 0.012) (Table 5). Eight (4.8%) NEC cases received a blood transfusion within 72 h of the diagnosis compared to one control (0.5%). The likelihood of having NEC was also 2.3-fold higher in neonates who were exclusively fed formula than those who were exclusively fed breastmilk (OR 2.32; 95% CI 1.37–3.93), and 2.7-fold higher in those fed breast milk with fortifier than without fortifier (OR 2.69; 95% CI 1.09–6.66; *p* = 0.032).

**Table 5:** Comparing feeding, use of antibiotics, and in-hospital management between the NEC cases and the controls with analyses of risk factors using logistic regression.

Risk factor	Cases	Controls	Univariate analysis	*P*-value	Multivariate analysis	*P*-value
	*N* = 144^a^	*N* = 206	Crude odds ratio		Adjusted odds ratio	
					(95% CI)	
	*n* (%)	*n* (%)	(95% CI)			
Feeding patterns						
Age at initiation of feeding, days	2 (2–3)^b^	3 (2–3)^b^	0.97 (0.87–1.08)	0.179	N/A	
Days to full enteral feeding	9 (6–12)^b^	10 (8–12)^b^	0.94 (0.88–1.00)	0.064	0.88 (0.80–0.95)	0.003
Exclusively fed formula vs. fed any amount of breastmilk						
Exclusively fed formula (*n* = 109)	56 (40)	53 (26)	1.92 (1.21–3.05)	0.005	2.00 (1.20–3.33)	0.008
Breastmilk or mixed feeding (*n* = 237)	84 (60)	153 (74)	Reference		Reference	
Management of the cases (before diagnosis) and controls						
Blood transfusion before diagnosis of NEC^c^	8 (5.6)	1 (0.50)	12.4 (1.53–100)	0.018	27.39 (2.09–359)	0.012
Diagnosed with patent ductus arteriosus	10 (6.9)	7 (3.4)	2.12 (0.79–5.71)	0.137	N/A	
Duration of antibiotic exposure before diagnosis of NEC/recruitment, days	3.5 (3–5)^b^	3 (2–4)^b^	1.18 (1.08–1.29)	<0.001	1.26 (1.14–1.40)	<0.001

^a^ Four patients were not fed prior to NEC diagnosis.

^b^ Median (interquartile range).

^c^ Small sample size, therefore wide confidence intervals.

### Comparing survivors and non-survivors amongst neonates with NEC

In the univariate analysis, the factors associated with mortality among the neonates with definite NEC were Modified Bell's stage 3 disease (*p* < 0.001), the need for ventilation (*p* < 0.001), and culture-confirmed sepsis (*p* < 0.001) (Table 6). In the multivariate logistic regression analysis, after adjusting for birth weight, modified Bell's staging, age at diagnosis, ventilation, hypotension, and sepsis, the factors that were associated with mortality were the need for ventilation, with a 6-fold increased likelihood of mortality (OR 6.23; 95% CI 2.23–16.8; *p* < 0.001), and hypotension requiring inotropes, with a 2-fold increase in the likelihood of mortality (OR 2.59; 95% CI 1.04–6.47; *p* = 0.04).

**Table 6:** Predictors of mortality among neonates with NEC in univariate and multivariate logistic regression models.

Variable	Non-survivors	Survivors	Univariate analysis	*P*-values	Multivariate analysis	*P*-values
	*N* = 80	*N* = 87	Crude odds ratio		Adjusted odds ratio	
	*n* (%)	*n* (%)	(95% CI)		(95% CI)	
Gestational age (weeks)	31.0 (29.0–33.0)^a^	31.3 (30–33)^a^	0.97 (0.87–1.07)	0.537	N/A	N/A
Birth weight (g)	1,508 (1,175–1,767)^a^	1,375 (1,165–1,695)^a^	1.00 (0.99–1.00)	0.254	N/A	N/A
Modified Bell's stage 3	51 (77.3)	15 (22.7)	8.44 (4.11–17.32)	<0.001	2.19 (0.89–5.4)	0.087
Sex, male	50 (62.5)	45 (51.7)	0.64 (0.35–1.19)	0.161	N/A	N/A
Age at diagnosis	7 (6–13)	11 (5–19)	0.98 (0.96–1.00)	0.141	N/A	N/A
Ventilation	71 (88.8)	28 (32.2)	16.62 (7.27–37.99)	<0.001	6.17 (2.28–16.70)	<0.001
Hypotension	48 (60)	12 (13.8)	9.38 (4.40–19.96)	<0.001	2.59 (1.04–6.47)	0.041
Sepsis^b^	57 (71.2)	34 (39.0)	3.86 (2.02–7.39)	<0.001	1.40 (0.61–3.21)	0.426

^a^ Median (interquartile range).

^b^ Sepsis was defined as culture-confirmed sepsis.

## Discussion

The overall incidence rate of NEC in this study was 3.8 per 1,000 live births and 4.5% amongst VLBW infants. Formula feeding, blood transfusion, and a longer duration of antibiotic exposure were identified as risk factors. The rate of culture-proven sepsis was high, and a substantial proportion of the NEC cases required surgical intervention. There was a high mortality rate, with infants of a lower gestational age more likely to die.

The incidence rates amongst the VLBW infants reported in this study differ from the 2%–7% reported from some high-income countries (HICs) (13, 18). A study from the Netherlands reported an increasing incidence rate of NEC among extremely preterm infants, as the gestational age for active medical intervention was decreased from 26^+^ to 24^+^ weeks (19). Studies in HICs that report a higher incidence of NEC than we have reported in this article have reported on very preterm infants born before 28 weeks and on extremely low birth weight infants. However, due to limited resources, advanced medical care is not offered to extremely preterm infants at CHBAH; hence, their survival rates are very low. The surviving preterm infants are more mature and of higher weight and therefore have a comparatively lower incidence of NEC than that of extremely preterm infants. In view of these differences in the patient populations, comparison with HICs is difficult. Other tertiary centers in South Africa have reported the incidence rate among VLBW infants to be 6%–8% (20, 21). This difference in the survival rates of extremely preterm infants could be attributed to the variations in weight cutoffs for advanced medical care in the different centers.

This study showed that prolonged antibiotic exposure was a risk factor, as previously shown by a retrospective study conducted in Kenya (22). It is postulated that antibiotic exposure causes microbial dysbiosis and thus predisposes the infant to NEC (23). A prospective study in a high-income country has previously shown that prolonged antibiotic exposure in preterm infants was a risk factor for NEC (24) and several retrospective studies have shown similar findings (25–27). However, a previous case-control study conducted at CHBAH did not find any association between antibiotic exposure and NEC. This difference could be due to the two studies being conducted approximately 15 years apart, and there have since been increased survival rates among extremely low birth weight infants (4).

This study found that any breastmilk was protective and reduced the risk of NEC by approximately 50%. Mother's own breastmilk contains Bifidobacteria and other flora, secretory IgA, and microbial proteins, which are protective. A prospective matched case-control study conducted in Europe by Berkhout et al. found that, after multivariate logistic regression analysis, the risk factors for NEC were formula feeding and a longer duration of parenteral feeding (24).

Antibiotic use and formula feeding can both cause disturbances in the microbiome. It has been postulated that resistant strains of bacteria may develop, and the integrity of the intestinal barrier may be compromised, with toll-like receptor 4 (TLR-4) activating the inflammatory cascade and leading to the development of NEC (28).

In this study, NEC was associated with a recent blood transfusion. Several mechanisms have been postulated. The anemia itself may lead to reduced oxygen delivery to the intestine and the resultant tissue ischemia may predispose the neonate to developing disease. Vascular tone is dependent on adequate levels of circulating nitric oxide. A blood transfusion may disrupt the expression of nitric oxide synthase, an important enzyme in the production of nitric oxide (29, 30). The depletion of nitric oxide results in increased vascular tone and, therefore, reduced blood supply to the intestinal mucosa, increasing the risk of NEC. Transfused blood contains broken red cell fragments, which can trigger the inflammatory process. Stored red blood cells may have high amounts of free hemoglobin, which may inactivate circulating nitric oxide and activate TLR-4, setting off an inflammatory cascade, which may contribute to the development of NEC (29, 31). Following a blood transfusion, the levels of IL-1β, IL-6, IFN-γ, and ICAM-1 increase, all of which may play a role in inflammation. There is conflicting evidence in the literature regarding the association between blood transfusion and NEC. Meta-analyses, such as those conducted by Mohamed et al. and Su et al., found that blood transfusions were associated with NEC (30, 32). However, other multicenter studies have failed to show a causal relationship (24, 31, 33). There is a lack of consensus and research regarding the association between blood transfusion and NEC is ongoing as this relationship and pathophysiology are not fully understood. There are controversies regarding whether feeds should be withheld or administered during a transfusion. Some authors believe that feeding during transfusion may predispose the neonate to the development of disease; however, the evidence is inconclusive (31). The association between NEC and blood transfusion observed in this study must be interpreted with caution as there was only one infant who received a blood transfusion in the control group, and the total number of neonates who were transfused was small, hence the wide confidence intervals.

The reported rate of sepsis among NEC cases varies between different tertiary hospitals in South Africa. In this study, the rate of culture-proven sepsis was 54.5%, which was higher than the 44% reported at Tygerberg Hospital in Cape Town, South Africa, but lower than the 71% reported at Charlotte Maxeke Johannesburg Academic Hospital (15, 16). These differences may be due to different background sepsis rates at these facilities. The background sepsis rates in the setting where this study was done are very high at 14.3/1,000 patient days compared to 3.3/1,000 patient days reported at Tygerberg Hospital (34, 35).

The NEC cases had a significantly slower weight gain after diagnosis compared to the controls. This has been attributed to functional gastrointestinal insufficiency in a previous study (36).

There was a high mortality rate of 47.9% among the cases, and the significant risk factors for mortality were ventilation and hypotension. The need for ventilation and the presence of hypotension requiring inotropes imply that the infants were sicker than those who did not need ventilation or inotropes. This mortality rate is similar to the 43.4% reported at a similar tertiary public hospital in Johannesburg (16). A retrospective study conducted in Cape Town showed a higher mortality rate of 52%, which may be due to the fact that the infants in that study were of a lower gestational age and birth weight, namely, 29 weeks vs. 33 weeks, and 1,185 g vs. 1,310 g, respectively. In that study, the significant predictors of mortality were lower gestational age, birth weight, and head circumference, as well as shock, apnea, and metabolic acidosis on admission (15). A retrospective study by Qian et al. found advanced disease, sepsis, and small for gestational age (SGA) to be risk factors for mortality (14). Other studies have found extremely low birth weight to be a risk factor for mortality (1). Studies from HICs have reported mortality rates between 21.4% and 28.8%, which are lower than the rate reported in this study. This difference is most likely due to more resources being available for management of these patients in HICs, and the fact that some of the studies reported mortality at 28 days, while this study reported mortality at final outcome or hospital discharge (24, 37).

The cases had a higher postnatal age than the controls. This most likely occurred by chance as the controls were enrolled toward the lower extreme of the 7-day limit used to match cases and controls. The controls who were older than the cases were more likely to have been discharged earlier, hence it was easier to find a control at the lower extreme of the 7-day limit.

The findings of this study highlight that NEC remains a burden in neonatal care of preterm infants in low and middle-income countries (LMICs) that is similar or worse than that observed in HICs, as evidenced by the high incidence rate of 4.5% in this study and 2%–7% in HICs. This burden of NEC is worse in LMIC settings as it is associated with high mortality of more than 40% compared to 23% in HICs (37). Though the risk factors observed in this study are similar to those previously reported in both HICs and LMICs, the rate at which they were observed in this study is relatively higher than that observed in HICs, suggesting that there is a lack of impetus to prevent this devastating disease. For example, only 53.3% of preterm infants in this study were exposed to antenatal steroids (cases: 55.9%; controls: 51.4%), despite antenatal steroids being known to reduce the risk of NEC by 50%, compared to HICs, where more than 80% of preterm infants are exposed to ANS (38). Second, a large proportion of preterm infants are still being exclusively fed formula feeds, despite the known benefits of breastmilk in preventing NEC. Third, there was high use of antibiotics among the cases, highlighting the importance of antibiotic stewardship, especially in the management of preterm infants. This study also reports a higher blood-culture positivity rate than that reported in HICs, with most of the positive cultures being Gram-negative. Infections have been implicated in the pathogenesis of NEC; however, infections could also be due to NEC itself as the integrity of the intestinal wall is compromised due to inflammation caused by NEC. Thus, it is not clear whether, in a setting where sepsis rates are high, such as in our setting, infection is the cause of NEC or rather a complication of NEC. The high blood-culture positivity rate suggests that infections may play more of a role in the development of NEC in LMICs than in HICs. Therefore, the strict implementation of infection prevention and control measures to prevent nosocomial infections may play a significant role in preventing NEC in LMICs. In summary, this study’s findings highlight that while the pathogenesis of NEC may be due to both intrinsic and extrinsic factors and the risk factors are similar in both LMICs and HICs, the extrinsic factors may play a more significant role than intrinsic factors in LMICs compared to HICs.

### Strengths and limitations of this study

The main strength of this study was the fact that it was a prospective study in an LMIC; therefore, a large amount of information was collected, including maternal and infant baseline characteristics and information relating to NEC diagnosis, management, and outcomes. This was a case-control study, which enabled comparisons between infants with and without disease. There are few prospective case-control studies on NEC in LMICs; therefore, this research adds valuable information to the body of knowledge on this condition.

A limitation is that the present study was conducted at a single center; therefore, the findings may not be generalizable to other populations. Due to resource limitations in the Radiology Department, the interpretations of the x-rays were conducted by the clinicians who were managing the infants, with no input from radiologists. This may have led to an overestimation or underestimation of the incidence of NEC by the clinicians, thereby potentially introducing bias.

The required number of controls (288) was not achieved due to time constraints, as the time needed to obtain consent from the mothers of infants competed with the researcher's clinical responsibilities.

## Conclusions

This study highlights that NEC remains a relatively common morbidity in neonates, particularly in VLBW infants. Formula feeding remains a significant risk factor for the development of NEC, and, therefore, exclusive breastfeeding should be prioritized as an important preventative strategy. Strategies to improve breastfeeding rates should include establishing a culture of exclusive breastfeeding in our communities and setting up donor milk banks. The second major risk factor was a blood transfusion. The mechanisms by which a blood transfusion is associated with NEC need to be studied further, as it is unclear whether this is a causative factor or not. Prolonged antibiotic exposure is another important risk factor; therefore, antibiotic stewardship should be practiced, and unnecessary use of antibiotics should be avoided. Addressing and preventing the causative factors of NEC should be one of the focus areas in managing neonates to reduce neonatal deaths.

## Data Availability

The original contributions presented in the study are included in the article/Supplementary Material, further inquiries can be directed to the corresponding author.
